# Heat Transfer Performance in a Superheater of an Industrial CFBC Using Fuzzy Logic-Based Methods

**DOI:** 10.3390/e21100919

**Published:** 2019-09-20

**Authors:** Jaroslaw Krzywanski

**Affiliations:** Faculty of Science and Technology, Jan Dlugosz University in Częstochowa, Armii Krajowej 13/15, 42-218 Czestochowa, Poland; j.krzywanski@ujd.edu.pl; Fax: +48-343615970

**Keywords:** heat transfer, superheaters, fluidization, steam boilers, fuzzy logic, artificial intelligence

## Abstract

The heat transfer coefficient in the combustion chamber of industrial circulating flidized bed (CFB) boilers depends on many parameters as it is a result of multifactorial mechanisms proceeding in the furnace. Therefore, the development of an effective modeling tool, which allows for predicting the heat transfer coefficient is interesting as well as a timely subject, of high practical significance. The present paper deals with an innovative application of fuzzy logic-based (FL) method for the prediction of a heat transfer coefficient for superheaters of fluidized-bed boilers, especially circulating fluidized-bed combustors (CFBC). The approach deals with the modeling of heat transfer for the Omega Superheater, incorporated into the reaction chamber of an industrial 670 t/h CFBC. The height above the grid, bed temperature and voidage, gas velocity, and the boiler’s load constitute inputs. The developed Fuzzy Logic Heat (FLHeat) model predicts the local overall heat transfer coefficient of the Omega Superheater. The model is in good agreement with the measured data. The highest overall heat transfer coefficient is equal 220 W/(m^2^K) and can be achieved by the SH I superheater for the following inputs l = 20 m, t_b_ = 900 °C, v = 0.95, u = 7 m/s, M-C-R = 100%. The proposed technique is an effective strategy and an option for other procedures of heat transfer coefficient evaluation.

## 1. Introduction

Since heat transfer processes are irreversible, some entropy accomplished by exergy destruction is generated [1]. These irreversibilities should be reduced to increase engine performance. One of the ways leading to an increase in a system’s efficiency is an analysis and optimization of heat transfer processes [2,3,4,5,6].

The overall heat transfer coefficients in heat exchangers, including heat transfer coefficients from bed to a heating surface incorporated into a furnace of a circulating fluidized bed combustor (CFBC) can be determined via various approaches. Detailed measurements, models, and correlations are mostly employed methods of heat transfer coefficients evaluation [7,8,9,10,11,12,13]. Three different tubular type instruments (flux tubes) were developed to identify steady-state boundary conditions in water wall tubes of steam boilers [10,11]. The authors provided detailed guidance which allows dealing with the uncertainty in determined parameters. A measurement procedure of the heat transfer coefficients during air- and oxy-firing conditions in the 90 kW oxy-fuel bubbling fluidized bed was shown in [12]. Maximum errors of 18% were reported in oxy-firing conditions. Sun et al. [13] studied the influence of crushed biomass pellets addition on bed-to-wall heat transfer in a 0.2 MW pilot-scale circulating fluidized bed (CFB) furnace. Fourteen K-type and differential pressure transmitters were used during the study. Bituminous coal and sawdust pellets were considered during the co-combustion tests. The authors observed a higher bed-to-wall heat transfer coefficient for higher biomass shares in the fuel. Dutta and Basu carried out experiments of heat transfer on wing-wall in a circulating fluidized bed pilot plant [14]. The investigations were limited to two positions at which the wing walls were hung.

Measurements are tedious and costly technics of the data acquisition process. In the context of experimental research, a question sometimes appears whether obtained results are still valid when the boiler’s operational parameters change and whether in such situations is it essential to carry out another experiment [15].

Multiple models and correlations constitute another way of heat transfer coefficient evaluation [2,3,4,7,16,17,18,19,20,21,22,23]. Thermodynamic second law analysis of heat transfer surfaces can be found in [2,3,4]. Detailed data, experimental or theoretical, are necessary to perform such calculations. Abdelmotalib et al. [9] distinguished four groups of heat transfer models: (a) film model, (b) unsteady state packed model, (c) discrete particle model, and (d) gas convection model. The authors underlined that discrete particle models have the potential to simulate gas flow and heat transfer at the same time. These kind of models consider a gas film between the surface and particles and includes the single-, two-, and four-particle models [9]. Basu and Nag presented a critical review of the existing mechanistic models allowing to describe the nature of heat transfer and the particle convective heat transfer component. The authors underlined a large variation in reported data between large- and laboratory-scale units, mainly due to uncertainties in the measurement of suspension densities in large-scale CFB boilers. They also pointed out that mechanistic models are no better than the hydrodynamic models on which they are based [7,16]. Molerus developed predictive equations for heat transfer in fluidized beds [24,25,26]. The applied dimensional analysis to identify seven relevant groups was based on the physical properties of the solids and gas. However, the approach does not consider radiation effects. A mechanistic heat transfer model, based on the cluster renewal approach, between a fluidized bed of group b particles and vertical rifled tubes has been presented in [17]. Maximum relative error was lower than 18.7%. 

It must also be noted that numerous correlations existing in the literature allow for estimating heat transfer coefficients only within a limited range, which restricts both their accuracy and generality [16,27]. Mechanistic models are constrained by the assumptions on which they were based whereas the empirical models have the accepted accuracy only within the range of experimental data on which they are built [9].

A correlation of the wall average heat transfer coefficient for six boilers in the range from 12 MW_th_ to 300 MW_th_ was presented in [28]. The root mean square deviation between measurements and the correlation was 15%. A general correlation for maximum heat transfer to surface submerged in gas-fluidized beds was provided in [8]. The author predicted 363 data points from 53 sources. The following range of parameters was included: heat transfer surface diameter 0.05–220 mm, particle diameter 31–15,000 µm, pressure 0.026–0.95 MPa, temperature 13–1028 °C. A mean absolute deviation was 16.2%. 

Computational fluid dynamics methods are powerful tools to predict heat transfer in fluidized bed [9,20,29,30,31]. Two main categories of CFD fluidized bed models can be distinguished: the Eulerian–Lagrangian approach, considering the gas as a continuum and tracks the particles through the gas flow field and the Eulerian–Eulerian two-phase models, where gas and each solid phase are considered to be as a continuum [9]. Chang et al. used Fluent software for predicting of particle–particle heat transfer between particle classes in a dense gas-solid fluidized bed in [20]. The simulations were performed up to 15 s of real-time due to the time step of 0.001 s. A thermal analysis of a tube ‘‘double omega’’, used in the steam superheaters in CFB boilers, was performed in [21,32]. Abdelmotalib et al. remarked that due to very complicated processes, which occur in the fluidized bed combustors, it is difficult to cover all aspects of heat transfer [9]. Therefore some additional, auxiliary data, e.g., to adjust parameters or to solve complex differential equations are necessary, and even other assumptions should be made to get a trackable solution [33,34,35,36,37,38]. As an example, Zhou et al. pointed out that simulations conducted on the platform of FLUENT 12 software with three processors parallel for 70 s with the number of meshes nearly 8500 costs nearly 60 days [15,39].

Finally, according to a recommended method for estimating total heat transfer in large–scale CFB boilers, Yang et al. noted, that the deviations of 25% between experimental and predicted heat transfer coefficient is acceptable [27].

Alternative methods for the above time-consuming and expensive techniques of data handling is the use of artificial intelligence (AI) approach. One of their representatives are called the neurocomputing routines [40,41,42,43], but there are also other AI techniques of data handling, including fuzzy logic (FL) approach [15,44,45,46]. The first one is based on the use of artificial neural networks, as they have the abilities to reproduce a process from training samples. A large enough set of training data is necessary to develop a neural network model, which is considered as one of the main disadvantages of these methods [15].

From the above literature review, an important question appears about the existence of an effective modeling method which is capable of developing a model providing accurate and quick results within a reasonable time. The answer seems to be a fuzzy logic-based approach. The method employs linguistic variables and is capable of describing vague, ill-defined, and complex issues. Fuzzy systems theory can also have utility in assessing some of more conventional, less sophisticated systems, e.g., when a fast solution can be useful in making preliminary operational or design decisions, to save computational costs, or where the inputs to a problem are vague, ambiguous, or not known at all [47]. Such a case exists, e.g., when dealing with CFB units, as they require fast control of temperature [9]. The method does not need a large amount of data and allows for formalizing a practical problem using the experience rather than the proper knowledge of the system. It is essential when dealing with large-scale CFB boilers, where many experimental data are hardly available. According to the literature, fuzzy systems are universal approximators and can handle a wide range of information. Hence, fuzzy systems have a high potential to understand complex, nonlinear systems, or problems with incomplete and inconsistent information [47]. 

On the other hand, fuzzy models can be considered as shallow models in the sense that they are primarily used in deductive reasoning, where we infer the specific from the general. These systems do not describe the underlying processes by which the observed data are generated, and this is the main limitation of the method [47,48].

The paper deals with the fuzzy logic-based method to determine the overall heat transfer coefficient of the Omega Superheater (SH I) in an industrial circulating fluidized-bed combustor. The novelty of the paper is an application of a fuzzy logic system for optimizing heat performance of the SH I, and this is the main contribution of the manuscript to the literature. This interesting approach uses limited data from experiments and provides quick and accurate results which allow for selecting optimal parameters of the CFB combustor for optimization of system efficiency. The presented non-iterative and effective approach allows carrying out a heat transfer performance analysis in the superheater and can be applied for any kinds of superheaters in all types of fluidized units, over various operating regimes. The developed model constitutes a new tool to select the optimal operating conditions of a CFB boiler.

## 2. Materials and Methods

**Figure 1:**
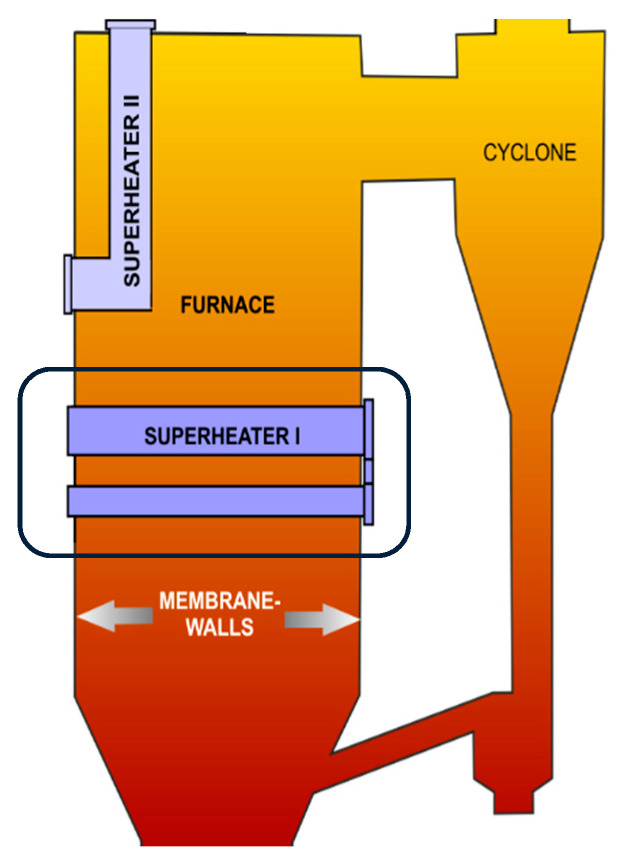
Superheaters in the furnace of the 670 t/h CFB boiler.

The computational study of the impact of control variables on the overall heat transfer coefficient for the Omega Superheater (Superheater I, SH I) was performed on the 235 MW_e_ circulating fluidized-bed boiler, operated in Turow Power Station, Poland (Figure 1). It is a commercial CFB combustor of 48 m height with natural water circulation and cross-sectional area of 21.2 × 5.2 m and 21.2 × 9.9 m, at the grid level and the height of 6.7 m above the grid, respectively. 

The Omega Superheater (SH I) is a superheater made of tubes spanning horizontally across the furnace, incorporated into the lower part of the combustion chamber [43,49] (Figure 1). The principal purpose of the heating surface is to excavate the additional heat for steam production. Since the bed in the region, where the Omega Superheater is located, has relatively high solids concentration, this heating surface works at severe operating conditions [21,50,51]. 

The SH I consists of 7 horizontal bundles of U-tubes, located in the distance 1.056 m of each other. Two sections can be distinguished in each bundle, the top section and bottom one, separated with the vertical gap of 1.064 m. 

The top and bottom sections contain 15 and 32 tubes of 0.051 m and 0.0445 m in diameter, respectively, and are arranged in the distance of 0.0635 m. The total surface of the Omega Superheater equals 770 m^2^.

**Figure 2:**
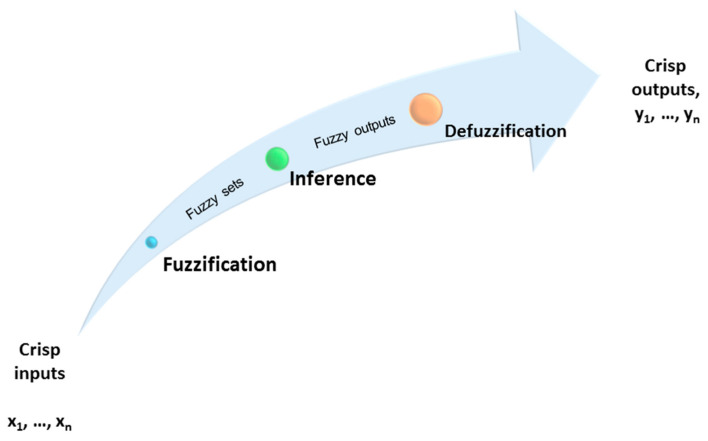
The scheme of a general fuzzy-logic model.

Experiments campaigns, previously carried out on the 670 t/h CFBC operating at the PGE Turow Power Plant in Poland, provided data allowing the FL model to perform [49]. The fuzzy-logic approach belongs to the AI methods which apply linguistic variables and fuzzy sets, capable of describing the behavior of a system [52]. Three principal stages of the Fuzzy Logic Heat (FLHeat) model’s development procedure can be distinguished: fuzzification process, inference procedure, and defuzzification operation (Figure 2).

In the fuzzification process, the entire domain of inputs is covered by the fuzzy sets, and a numeric value of an input is assigned a membership function from the range between 0 and 1.

The membership function of a fuzzy set is a function which maps each crisp element of a numerical domain into an interval [0,1] [15].

During the next step. i.e., the inference stage, fuzzy outputs are generated using the fuzzy rule-base, also called the IF-THEN rule-base. The typical IF-THEN rule-base for inputs x_1_ and x_2_, membership functions M_1_ and M_2_, and the output y may be described, as:

IF x_1_ is M_1_ and i_2_ is M_2_ THEN y is y = m (x_1_, x_2_) (1)

where m (x_1_, x_2_) is a polynomial function of the inputs x_1_ and x_2_ [53,54,55].

Finally, after accumulation operation, when the results of individual rules are combined, during the defuzzification process, the crisp outputs are generated [47,54,55]. A detailed description of the fuzzy logic procedure can be found elsewhere [15,47]. 

An essential feature of this method is the fact that the FL approach allows for describing a process on the base of experiences. Proper knowledge of a system is not required. 

For this work, the Qtfuzzylite (http://www.fuzzylite.com) application, which is also a fuzzy logic control tool, has been used. The validation procedure of the developed FLHeat model was successfully carried out against data of the heat transfer coefficients for the Omega Superheater obtained during experiments [55]. 

Five inputs are applied to perform the FLHeat model: the height above the grid l, bed temperature t_b_ and voidage v, gas velocity u and the boiler’s load M-C-R of the industrial-scale CFB unit. Such selected inputs allow for describing the overall heat transfer coefficient k for Omega Superheater, as the output parameter [50]. All inputs and output are described in Table 1.

**Figure 3:**
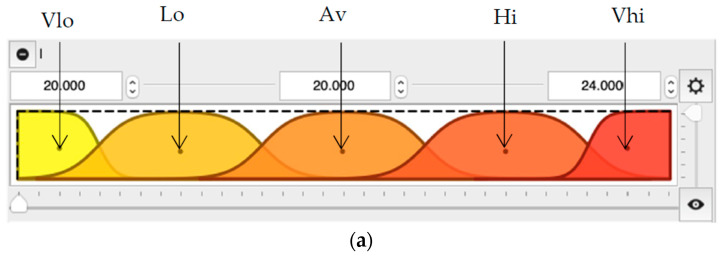

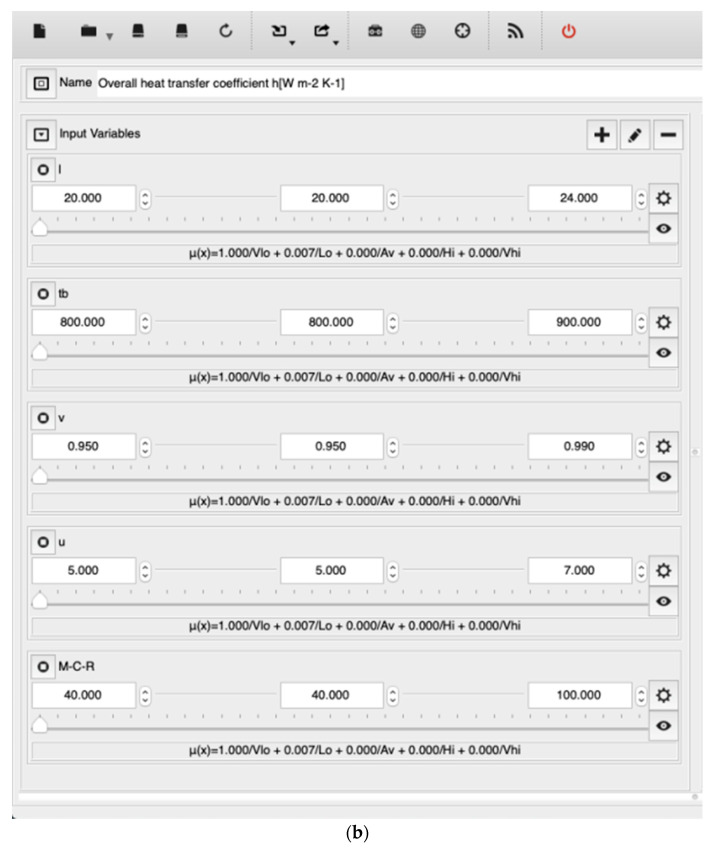

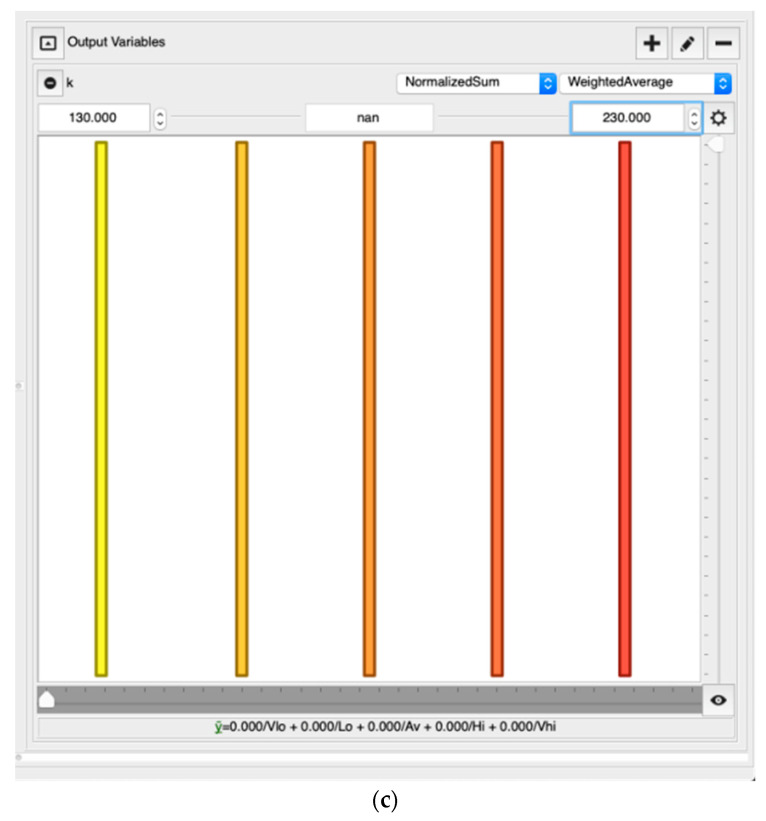
The FLHeat model; (**a**) a graphical representation of sigmoid linguistic terms of inputs (l constitutes an example), (**b**) inputs of the model, (**c**) graphical representation of the output value.

The FLHeat model uses sigmoid and constant terms for describing the inputs and output parameters, respectively. Five linguistic terms were determined: Vlo (very low), Lo (low), Av (average), Hi (high), and Vhi (very high). Figure 3 depicts the inputs and output parameters of the developed FLHeat model.

Such established fuzzy sets allow for assigning a vector of numeric input values to a vector of membership degrees, i.e., fuzzy sets M [47]:

(2) M= [µ(Vlo)/Vlo +µ(Lo)/Lo + µ(Av)/Av+µ(Hi)/Hi+µ(Vhi)/Vhi]

Table 2 defines the IF-THEN rule-base. Fuzzy output was generated via the Takagi–Sugeno method [47,53].

The weighted average defuzzification method of crisp values calculation, expressed by the Formula (3), is employed during the defuzzification stage [47]:

(3) $$y=\;\sum µ\left(\bar{y}\right)\bar{y}/µ\left(\bar{y}\right)$$

where $\bar{y}$ are centroids of membership functions.

Such developed FLHeat model allows for studying the effects of input parameters on the heat transfer coefficient of the SH I superheater as well as the selection of optimum operation strategies of the CFB boiler. This non-iterative method allowed for obtaining results immediately, so fast computations are the main advantages of the FLHeat model.

**Figure 4:**
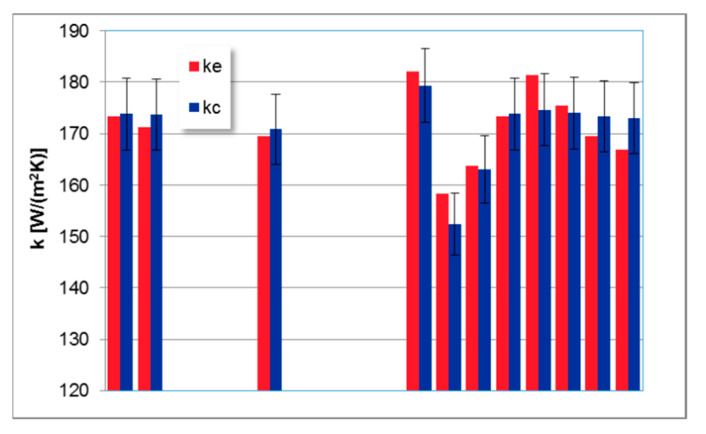
Comparison of heat transfer coefficient for the Superheater I desired (ke) and predicted (kc) by the FLHeat model.

The developed model has been successfully validated on the desired (obtained from experiments) data (Figure 4). Bars of errors depicted in Figure 4 correspond to 4% of the relative error between desired and predicted data. The accuracy of the model is reasonable, and the developed FLHeat model gives good predictions since the deviations of 25% between predicted and experimental data of heat transfer are considered as acceptable for large commercial CFB units [27].

It means that the accuracy of the FLHeat model is good enough to be used for predicting heat transfer coefficients in the SH I superheater.

## 3. Results and Discussion

The overall heat transfer coefficient is described by a sum of three components: i.e., (i) gas convective heat transfer coefficient, (ii) particle convective heat transfer coefficient, and (iii) radiative heat transfer coefficient [9,56,57,58]. These three components allow for in-depth analysis when discussing the influence of operating parameters on the overall heat transfer coefficient. 

**Figure 5:**
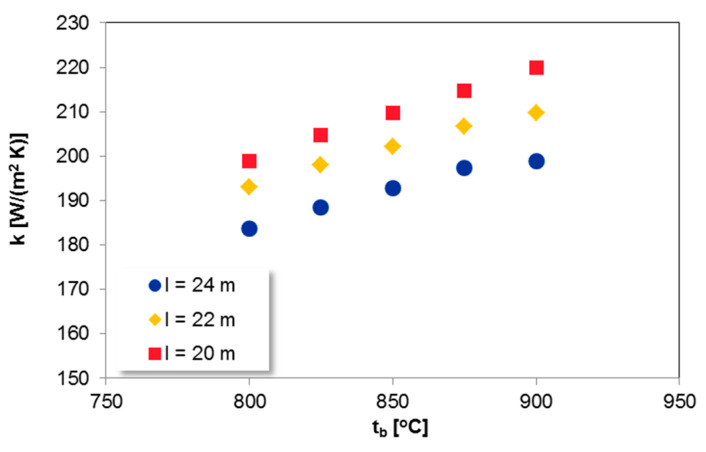
The influence of t_b_ on heat transfer coefficient k for SH I; u = 7 m/s, v = 0.95, M-C-R = 100%.

Figure 5 shows the effect of the bed temperature t_b_ on the heat transfer coefficient k.

The increase in t_b_ causes the rise in k due to higher radiation and thermal conductivity of gas at higher temperatures [7,59,60]. Although the considered Omega Superheater is located in the fast bed, the average suspension density of the bed is relatively high, equal around 20 kg/m^3^. Therefore, the calculated profiles, given in Figure 5 are almost linear as for the dense beds.

**Figure 6:**
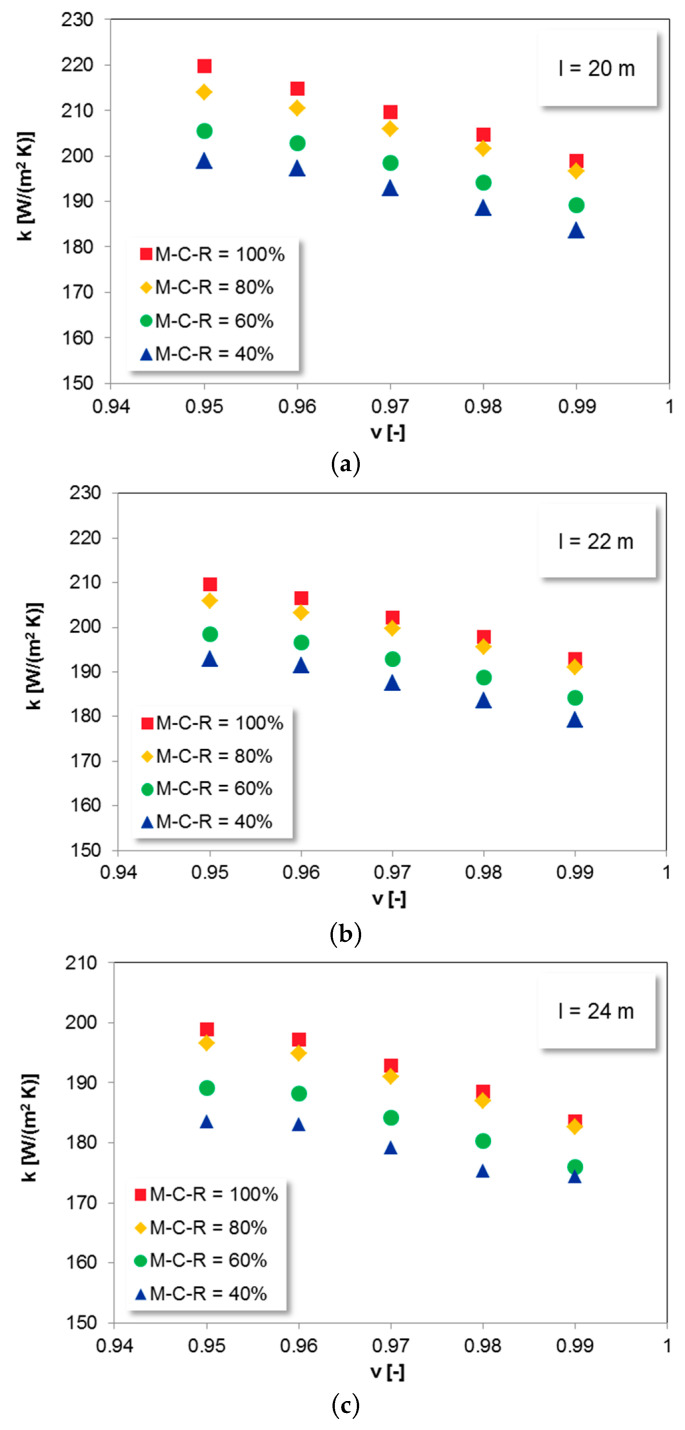
The influence of v on heat transfer coefficient for SH I; u = 7 m/s, t_b_ = 900 °C.

The influence of bed voidage on the overall heat transfer coefficient is shown in Figure 6. As the bed voidage increases the heat transfer coefficient decreases.

The bed voidage is considered as the volume fraction of the bed occupied by bubbles. So, the increase in bed voidage of a bed volume means the decrease in solids concentration in the volume. 

Breitholtz et al. underlined that particle convection is the dominant convective component in heat transfer from bed to the heating surface due to higher density of the particles and their volumetric heat capacity than that of the gas [28]. Therefore, the presence of solid particles and their higher concentration leads to the increase in heat transfer rates. According to Ebert et al. in the range of suspension densities between 12 and 80 kg/m^3^ the particle convection dominates, and the gas convection component corresponds to 10%–20% of the total heat transfer [16,61].

Furthermore, considering the heat transfer mechanism between particles and a wall the contact-time as well as the contact area between particles and the surface are small and thermal conductivity of the gas determines the heat transfer between the gas-particle suspension and the wall [28]. Since the thickness of this particle-free gas-gap increases with a decrease in the local suspension density, the increase in the average bed voidage will result in the decrease of k [7,16,28,51,62,63,64,65,66] (Figure 6).

The reported results from Figure 6 also reveal that the total heat transfer rate increases on the way down of the Omega Superheater, for lower tubes, where the bed material cannot reach the thermal equilibrium with the heating surface [7,14,27]. As the solids move up along the surface, their temperature gradient decreases as they get cooled, leading to a decrease in the heat transfer coefficient. That is why the local heat transfer coefficient increases towards the bottom tubes of the SH I.

**Figure 7:**
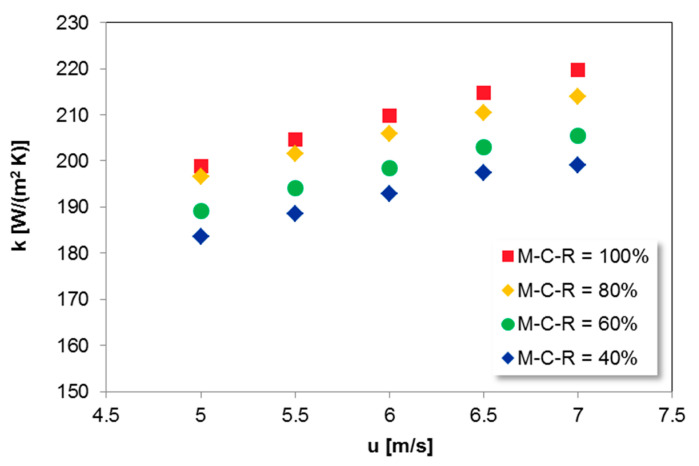
The influence of u on the heat transfer coefficient for SH I; t_b_ = 900 °C, v = 0.95, l = 20 m.

The increase in gas velocity generates higher heat transfer coefficients reported in Figure 7. In commercial boilers, primary air velocity has the main effect on the SH I heat transfer coefficients as more solids flow to upper parts of the combustion chamber. It is the reason why the proper design of air nozzles in CFB units is a fundamental issue [49,67,68].

Higher gas velocities result in both higher suspension densities and Reynolds numbers in the SH I region of the CFB furnace. As the two main components of the overall heat transfer coefficient increase, i.e., gas and particle convective heat transfer coefficients, the total heat transfer coefficient also increases.

**Figure 8:**
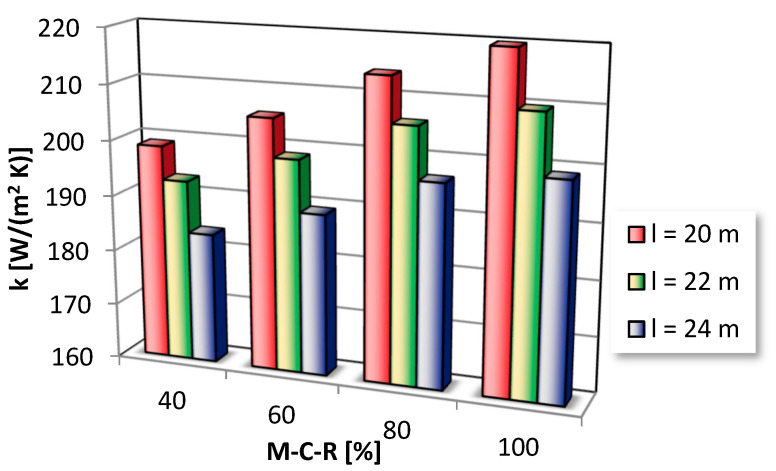
The impact of the load of the boiler; t_b_ = 850 °C, u = 7 m/s, v = 0.95.

The influence of the load of the boiler on the overall heat transfer coefficient is depicted in Figure 8. Heat transfer mechanisms are intensified with loads of the CFBC, causing the growth in heat transfer coefficient k. It is mainly due to favorable hydrodynamic and thermal conditions which occur in the vicinity of the SH I. 

The solids behavior and thermal conditions are different for various loads. Higher gas velocities, solids circulating rates, and bed temperatures corresponding to higher M-C-R lead to the increase in heat transfer coefficients. 

It is also worth noting that the increase in M-C-R is bound not only with an increase in the bed temperature, gas velocity in the combustion chamber, and solids circulating rate, but also with a change in other boiler operating parameters, such as primary and secondary air distribution, suspension density profile in the furnace. Therefore, the use of M-C-R as an input makes the FLHeat model more complete and able handling other vital factors influencing the heat transfer coefficient, which are usually ill-defined and uncertain or difficult to measure or sometimes even hard to identify.

The optimization issue concerning the maximum heat transfer coefficient can be performed for the ranges from Table 1, using the developed FLHeat model. Calculations carried out in the paper revealed that the highest local heat transfer coefficient could be achieved by the SH I superheater for the following input parameters: l = 20 m, t_b_ = 900 °C, v = 0.95, u = 7 m/s, M-C-R = 100%. The highest value of k, possible to reach for the considered range of input parameters is equal to 220 W/(m^2^K).

The developed FLHeat model is an effective optimization tool, useful in matching optimum operating conditions of the large-scale CFB boilers.

## 4. Conclusions

The study deals with a novel application of a fuzzy logic system for optimizing heat performance of the Omega Superheater in an industrial-scale 235 MW_e_ CFB combustor. The calculations results obtained by the developed FLHeat model are in good agreement with the measured data. The relative, validation error of the prediction is lower than 4%.

The local heat transfer coefficient increases with bed temperature, gas velocity, and M-C-R, and decreases with the height above the grid and the bed voidage.

The highest overall, local heat transfer coefficient equal to 220 W/(m^2^K) can be achieved by the SH I superheater for l = 20 m, t_b_ = 900 °C, v = 0.95, u = 7 m/s, and M-C-R = 100%.

The developed FLHeat model may be regarded as a useful optimization tool. The possibility of obtaining results in a short time and their high accuracy are the primary advantages of the method. On the other hand, the availability of expert knowledge and a small number of input variables are the main limitations of this technique. Intelligent hybrid systems, combining fuzzy logic, neural networks, and genetic algorithms, may overcome these shortcomings.

## Figures and Tables

**Table 1:** Variables of the model.

Parameter	Values
l, m	20–24
t_b_, °C	800–900
v	0.95–0.99
u, m/s	5–7
M-C-R, %	40–100
k, W/(m^2^⋅K)	138–220

**Table 2:** The IF-THEN rule-base.

Heat Transfer Coefficient, W/(m^2^K)	Vhi	Hi	Av	Lo	Vlo
l, m	Vlo	Lo	Av	Hi	Vhi
t_b_, °C	Vhi	Ho	Av	Lo	Vlo
v	VLo	Lo	Av	Hi	Vhi
u, m/s	Vhi	Hi	Av	Lo	Vlo
M-C-R, %	Vhi	Hi	Av	Lo	Vlo

## Nomenclature

k	overall heat transfer coefficient, W/(m^2^⋅K)
kc	overall heat transfer coefficient, predicted (calculated), W/(m^2^⋅K)
ke	overall heat transfer coefficient, desired (measured), W/(m^2^⋅K)
x_i_	an i-th crisp input
M-C-R	maximum continuous rating, %
μ (x_i_)	a membership degree
y_i_	an i-th crisp output
t_b_	fluidized-bed temperature, °C
u	velocity, m/s
v	voidage
l	the height above the grid, m
b	fluidized-bed
